# Senescence and Apoptosis: Architects of Mammalian Development

**DOI:** 10.3389/fcell.2020.620089

**Published:** 2021-01-18

**Authors:** Emma Wanner, Harikrishnan Thoppil, Karl Riabowol

**Affiliations:** ^1^Department of Biology, Faculty of Science, University of Calgary, Calgary, AB, Canada; ^2^Department of Biochemistry and Molecular Biology, Cumming School of Medicine, University of Calgary, Calgary, AB, Canada; ^3^Department of Oncology, Cumming School of Medicine, University of Calgary, Calgary, AB, Canada

**Keywords:** apoptosis, development, Hox proteins, retinoblastoma, ING proteins, epigenetics, cancer, senescence

## Abstract

Mammalian development involves an exquisite choreography of cell division, differentiation, locomotion, programmed cell death, and senescence that directs the transformation of a single cell zygote to a mature organism containing on the order of 40 trillion cells in humans. How a single totipotent zygote undergoes the rapid stages of embryonic development to form over 200 different cell types is complex in the extreme and remains the focus of active research. Processes such as programmed cell death or apoptosis has long been known to occur during development to help sculpt organs and tissue systems. Other processes such as cellular senescence, long thought to only occur in pathologic states such as aging and tumorigenesis have been recently reported to play a vital role in development. In this review, we focus on apoptosis and senescence; the former as an integral mechanism that plays a critical role not only in mature organisms, but that is also essential in shaping mammalian development. The latter as a well-defined feature of aging for which some reports indicate a function in development. We will dissect the dual roles of major gene families, pathways such as Hox, Rb, p53, and epigenetic regulators such as the ING proteins in both early and the late stages and how they play antagonistic roles by increasing fitness and decreasing mortality early in life but contribute to deleterious effects and pathologies later in life.

## Mammalian Development

Mammalian development begins with the fertilization of the female gamete, the ovum, by a sperm cell, the male gamete, forming a single celled zygote. Following fertilization, the zygote undergoes rapid cleavage to form a solid ball of cells without cytoplasmic growth (Aiken et al., 2004). The next stage of development, blastulation, shows a series of morphological changes that result in the formation of a blastocyst consisting of an inner cell mass (ICM), outer trophectoderm cell layer, and a fluid filled blastocoelic cavity (Biggers et al., 1988). Gastrulation then ensues, which shapes the three germ cell layers; the endoderm, mesoderm and ectoderm, which during organogenesis develop into the tissues and organs of the developing organism (Solnica-Krezel and Sepich, 2012). The inner germ layer, the endoderm, gives rise to organs of the respiratory and digestive systems and to the epithelial cells that line the insides of these organs (Zorn and Wells, 2009). The middle mesoderm layer is responsible for the formation of connective tissues, blood and bone, the vascular system, muscles, the heart, the kidneys, and the reproductive organs (Kimelman, 2006). The outer ectoderm germ layer gives rise to the central and peripheral nervous systems, hair, skin, nails, the mouth, the nasal cavity, the lens of the eye and various exocrine glands (Sasai and Robertis, 1997). Neurulation, the formation of the neural tube from the folding in and fusion of the neural plate then occurs, which is essential for brain and spinal cord development (Copp et al., 2003).

Normal development occurs until sexual maturity at which point the slow process of biological aging occurs at varying rates in all cell, tissue and organ types. Aging has been described as a progressive deterioration of physiological functions resulting in increased risk of impairments, disease and mortality (Strehler, 1977). Genomic instability, Telomere attrition, epigenetic alterations, loss of proteostasis, deregulated nutrient sensing, mitochondrial dysfunction, cellular senescence, stem cell exhaustion, and altered intercellular communication have been described as “hallmarks of aging” and likely all contribute to the aging phenotype (López-Otín et al., 2013). Apoptosis has long been known to be essential for the formation and preservation of tissue patterning (Meier et al., 2000). Somewhat unexpectedly, senescence has been proposed to also play roles in development of the placenta (Rajagopalan and Long, 2012; Chuprin et al., 2013) and embryo (Davaapil et al., 2017), functioning mainly in tissue remodeling (Nacher et al., 2006; Muñoz-Espín et al., 2013; Storer et al., 2013; Zhao et al., 2018; Gibaja et al., 2019) and beginning early in evolution (Villiard et al., 2017), but how this occurs is not yet fully understood.

### Stem Cells in Development

Stem cells are defined by their ability of unlimited self-renewal and the capacity to divide into differentiated cells of developed tissues (Reya et al., 2001). Following fertilization, the zygote is composed of “totipotent stem cells,” meaning its cells can divide and differentiate into all cell types of the organism and extraembryonic tissues (Seydoux and Braun, 2006). Next, after blastulation occurs, the ICM develops the naïve epiblast which gives rise to “pluripotent embryonic stems cells” (ESCs) (Wu et al., 2016). Pluripotency is a property of cells that allows them to give rise to cells of all three germ layers of the developing embryo, but not the extraembryonic structures (Wu et al., 2016). Cells that give rise to particular germ layers are “multipotent stem cells” (Alison et al., 2002). Multipotent stem cells include hematopoietic stem cells (HSC), mesenchymal stem cells (MSC), and neural stem cells (Caplan, 1991; Bhatia et al., 1997; Gage, 2000). They can produce a limited set of differentiated cells of a specific tissue type or organ. “Unipotent stems cells” are only capable of dividing into a single stem cell type. For this reason, they are often called committed progenitor cells (Alison et al., 2002). Adult stem cells exist in different tissues throughout the mature mammal and remain undifferentiated, allowing them to help in tissue regeneration and replenishment (Wagers and Weissman, 2004). In mammals, a molecular mark of the totipotent cells that arise following fertilization is widespread DNA demethylation (Guo et al., 2014; Baker and Pera, 2018). Maintenance of pluripotent ESCs is governed and marked by the expression patterns of key transcription factors including OCT3/4, SOX2, NANOG, Klf4, and c-Myc (Nichols et al., 1998; Avilion et al., 2003; Chambers et al., 2003; Takahashi et al., 2007; Angeles et al., 2015).

### The Hox Gene Family Is Central to Mammalian Development

Hox gene family members are crucial to mammalian development. They code for transcription factors that determine the anteroposterior (AP) patterning of bilateral organisms during development (Garcia-Fernàndez, 2004). Vertebrates have 39 Hox genes, that due to gene duplications, are organized into four gene clusters, HoxA, HoxB, HoxC, and HoxD, on separate chromosomes (Lappin et al., 2006; Mallo et al., 2010). Hox genes are activated in a 3′ to 5′ direction, resulting in early developing structures having an anterior identity controlled by 3′ Hox genes, and later formed structures having a posterior identity developed mainly under the control of 5′ Hox genes (Forlani et al., 2003). During embryogenesis in mammals, Hox genes are expressed in all three germ layers, the endoderm, mesoderm and ectoderm (Krumlauf, 1994). The expression of Hox genes directs axial positioning of these developing embryonic tissues (Mallo et al., 2010).

The activation of Hox gene expression domains is controlled by processes that take place at the same time as the formation of the primitive streak during embryogenesis. Initiation of Hox gene transcription is controlled by signaling molecules including Wnt3, Wnt3a, FGF3, FGF8, and Retinoic acid (Bel-Vialar et al., 2002; Manzanares et al., 2002; Walshe et al., 2002; Weisinger et al., 2010; Schulte and Frank, 2013). Wnt signals regulate the formation and functioning of the primitive streak, and thus have been suggested to play a role in the forward anterior spreading of Hox gene expression (Forlani et al., 2003). Wnt signaling is required for expression of Hox genes involved in the developing nervous system of vertebrates. Specifically, Wnt3a signaling is important in hindbrain and spinal cord development through its role in Meis and Hox gene activation (Schulte and Frank, 2013). Similarly, FGF3 and -8 signaling molecule expression is present in the developing nervous system of all vertebrates, and PG2-3 Hox gene expression is dependent on FGF ligands (Walshe et al., 2002; Schulte and Frank, 2013). Retinoic acid (RA) is involved in regulating expression of PG1-4 Hox genes (Bel-Vialar et al., 2002). To function in embryonic development, Hox genes often rely on interactions with the Hox cofactors, TALE homeodomain proteins (Moens and Selleri, 2006). The vertebrate genome codes for the Meis/Prep and PCB TALE homeodomain protein families which are comprised of Meis1-3, Prep1-2, Tgif1-2, and Pbx1-4 proteins, respectively (Schulte and Frank, 2013).

Hox genes encode homeodomain transcription factors, that activate or repress target genes involved in vertebrate development by binding Hox-response enhancers (Pearson et al., 2005). Several Hox genes are involved in establishment of the distinct vertebral column segments from the mesodermal tissue along the neural tube (Deschamps and van Nes, 2005). Interestingly, *in vitro* in melanocytes, Hox10A has been shown to act with cofactors, Meis1 and PBX1 TALE homeobox transcription factors to activate Cdkn1a which encodes p21, leading to growth arrest, potentially connecting Hox proteins to apoptosis and/or senescence (Bromleigh and Freedman, 2000).

## Epigenetics in Mammalian Development

Epigenetics can be broadly described as the changes made to an organism’s hereditary material which result in alterations of gene expression without changes to the DNA sequence (Goldberg et al., 2007). There are several types of epigenetic regulation that affect gene expression, using covalent modifications of DNA or core histones to produce inactive heterochromatin or active euchromatin in mammals (Li, 2002). DNA methylation, the addition of a methyl group to a cytosine nucleotide by a DNA methyltransferase (DNMT) enzyme, usually results in gene silencing, due to transcriptional repression at the promoter region (Das and Singal, 2004). There are many inhibitors of DNMTs that effect the cell cycle. The drug Atorvastatin, inhibits cell proliferation and induces apoptosis rat vascular smooth muscle cells (VMCs) (Zhu et al., 2017). Atorvastatin inhibits DNMT1, reducing methylation in the p16 promoter, increasing the activity of this tumor suppressor (Zhu et al., 2017). The HMG box-containing protein 1 (HBP1) transcription factor, is a repressor of DNMT1 that results in cellular senescence (Pan et al., 2013). HBP1 has a dual transcriptional role in activating senescence, through binding and repressing DNMT1 promoter and binding to the activation element of p16 (Pan et al., 2013). The repression of DNMT1 and decreased methylation of p16 promoter also increases expression of p16, inducing senescence (Pan et al., 2013). The DNA methyltransferase inhibitors (DNMTis), 5-azacytidine and 5-aza-2′-deoxycytidine, have differing cellular responses in tumor cells (Venturelli et al., 2013). 5-azacytidine induces p53-dependent cell senescence indicated by an increase in senescence-associated marker β-galactosidase (SA-β-gal) senescence associated heterochromatin foci (SAHF), and induction of senescence-associated secretory phenotype (SASP) which are markers of senescent cells (Venturelli et al., 2013). However, 5-aza-2′-deoxycytidine induces apoptosis, through the activation of executioner caspases 3 and 7 and downregulation of p53 (Venturelli et al., 2013). Another form of epigenetic regulation is histone-post translational modification (PTM). The tails of histones that localize to the outside edges of nucleosomes can undergo various modifications such as acetylation, methylation, phosphorylation, ubiquitylation and sumoylation, among others (Bannister and Kouzarides, 2011). PTM’s help regulate chromatin structure, recruit other chromatin remodeling enzymes, and influence transcription, DNA repair, and replication. Substitution of the four core histones, H2A, H2B, H3, and H4, with histone variants changes the composition of chromatin and is an important epigenetic mechanism (Henikoff and Smith, 2015). Histone variants include CENP-A, H3.3, H2A.X, H2A.Z, macroH2A, and H2A.B. ATP-dependent nucleosome remodeling is another form of epigenetic regulation, in which ATP-dependent protein complexes use energy from ATP hydrolysis to change the interactions of histones with the DNA by relocating nucleosomes, thus altering gene expression (Vignali et al., 2000). The four families of SWI-like ATP-dependent chromatin remodeing complexes, SWI/SNF, ISWI, CHD, and INO80, reposition nucleosomes, making DNA more or less accessible for transcription (Ho and Crabtree, 2010). Short and long non-coding RNAs impose another form of epigenetic regulation that usually results in gene silencing (Peschansky and Wahlestedt, 2013). For example, *Xist* RNA, a long non-coding RNA is essential in X chromosome inactivation in females during mammalian development (Wei et al., 2016) and many different microRNAs affect the levels of numerous mRNAs. Epigenetic modifications have frequently been implicated in both apoptosis and senescence (Moskalev et al., 2014).

### The Epigenetic Clock of Aging and Effects on Development

The epigenetic clock of aging refers to findings that specific sites of DNA methylation generally negatively correlate with biological aging (Hannum et al., 2013; Horvath, 2013). Additionally, as organisms age, “epigenetic drift” occurs where methylation patterns are not stably maintained following repeated rounds of DNA repair and replication (Veitia et al., 2017). In mammals, DNA methylation patterns established by *de novo* and maintenance DNA methyl transferases (DNMT’s) are essential for embryogenesis (Goll and Bestor, 2005). Between zygote formation and blastocyst formation, both sets of chromosomes undergo demethylation, except imprinted genes whose methylation marks are preserved (Kafri et al., 1992). Following implantation, DNA methylation levels increase in the primitive ectoderm, which forms the whole embryo, but methylation patterns are not established in cells that form the extraembryonic tissues, the primitive endoderm and trophoblast (Chapman et al., 1984). The study done by Okano et al. (1999) demonstrated the importance of DMNT’s in embryogenesis as mice null for Dnmt3b, died between embryonic day 14.5 and 18.5 and showed multiple developmental defects. Although the closely related Dnmt3a was not found to be essential for early embryonic development, Dnmt3a null mice died in early development at around 4 weeks (Okano et al., 1999), highlighting the importance of such epigenetic mechanisms for development.

### Histone Post Translational Modifications (PTMs) in Development

Early stages of mammalian embryonic development entail development of the totipotent zygote into a nearly hundred cell blastocyst. The pluripotent inner layers of the blastocyst known as the trophoblast eventually give rise to all cell lineages of the fully matured mammal and have unlimited self-renewal (Wu et al., 2016). Following blastula formation, the naïve epiblast develops from the ICM, which gives rise to all pluripotent ESCs. The chromatin of pluripotent stems cells is mainly in an open euchromatin state resulting in transcriptional hyperactivity (Efroni et al., 2008; Gaspar-Maia et al., 2010). Pluripotency of stem cells during embryonic development is controlled by various epigenetic regulators. Histone PTM’s, DNA methylation patterns established by DNMTs, Histone methylation patterns established by histome methyl transferases (HMTs) and histone demethylases (HDMs), histone acetylation patterns through histone acetyltransferase (HAT) and histone deacetylase (HDAC) activity, and chromatin remodeling by ATP-dependent chromatin remodeling complexes all influence ESCs (Gaspar-Maia et al., 2010). Pluripotent ESCs have many epigenetic marks characteristic of active chromatin such as H3K4me and acetylation of H3 and H4, compared to differentiated cells (Gaspar-Maia et al., 2010). HDMs maintain the low level of H3K9me3 and H3K27me seen in undifferentiated ESCs (Gaspar-Maia et al., 2010). DNMTs establish methylation of CpG islands at promoter regions of repressed genes needed for differentiation of ESCs, making up around 30% of genes in these cells (Fouse et al., 2008). The ATP-dependent chromatin-remodeling enzymes CHD1 and esBAF and the TIP60-p400 complex maintain the open euchromatin state of ESCs and repress genes required for their differentiation and loss of pluripotency, respectively (Ho and Crabtree, 2010).

## Senescence

**FIGURE 1:**
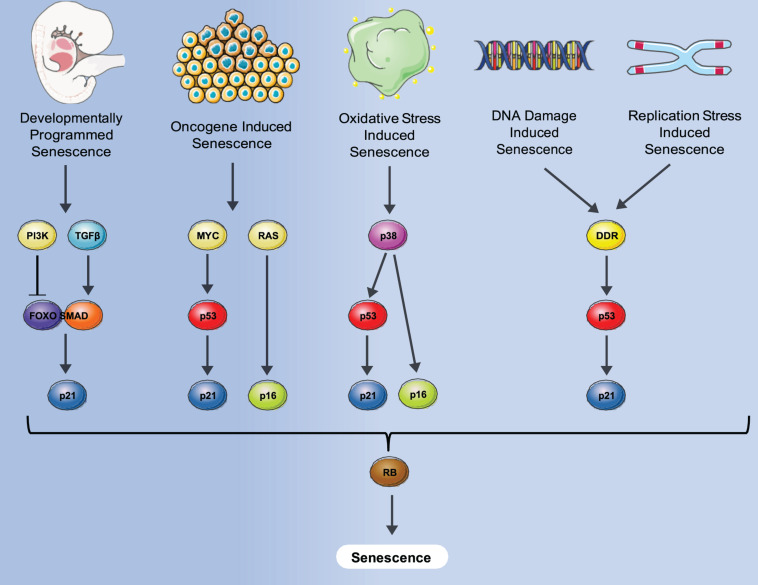
Different senescence induction methods and molecular pathways involved. A variety of stresses such as oncogene, oxidative damage, DNA damage, normal replication and development can induce senescence via either p53-induced expression of p21, p16 expression or both, converging to block Rb inactivation, and leading to senescence where an irreversible cell cycle arrest is imposed.

Senescence is defined as a phenomenon in which cells enter a state of permanent cell cycle arrest. The first observation of cell senescence was reported by Hayflick and Moorhead while trying to culture primary cells for an extended period of time for the purpose of making viral vaccines. They observed that primary human fibroblasts stop dividing after a certain number of passages that was variable, dependent on the particular donor (Hayflick and Moorhead, 1961) and described it as replicative senescence. Replicative senescence was later attributed to a decline in telomere length that occurred during both *in vitro* culturing and *in vivo* aging (Levy et al., 1992). Senescence was also found to be induced prematurely by activation of the ras oncogene in primary human cells which was negated by the activation of the p53/Rb/p16 pathways (Serrano et al., 1997). This type of senescence, termed oncogene-induced senescence (OIS) demonstrated the potential role of senescence as an anti-cancer mechanism designed to suppress proliferation of cells with oncogenic mutations. Senescence can also be induced by many additional stresses that affect numerous biological processes including oxidative stress, replication stress and notably DNA damage- induced stress shown in Figure 1.

Initially senescence was thought of only as a mechanism that contributed to various forms of morbidity associated with aging including immuno-senescence, impaired wound healing, impaired gastrointestinal function, and bone loss and frailty, *inter alia* (Strehler, 1977). While this remains widely accepted, a solely beneficial mechanism was later proposed in which senescence served as a first line of defense against cancers by limiting their growth (Campisi, 2003). However, very recent studies have also begun to link senescence with the emergence of cancer due to the effects of senescent cells on local tissue microenvironments through the secretion of a complex mixture of proinflammatory factors termed the senescence-associated secretory phenotype (SASP). The SASP consists of inflammatory cytokines, growth factors, chemokines, and proteases. While the SASP helps promote recognition and clearance of senescent cells by phagocytosis in the short term, long term secretion has a variety of age-related deleterious effects (Xue et al., 2007). In a landmark study, SASP from senescent cells co-cultured with tumor cells was shown to promote tumor progression (Coppe et al., 2008). Moreover, a cascade effect involving prolonged exposure to SASP inducing senescence in nearby fibroblasts via an ROS/DNA damage mechanism has also been reported (Nelson et al., 2012). Soluble signaling factors are major components of the SASP, the most prominent of which is interleukin-6 (IL-6). Recent evidence suggests a role of the GMP-AMP synthase (cGAS)-stimulator of interferon genes (STING) in SASP regulation. This is initiated by recognition of senescence induced cytosolic DNA by cGAS which in turn activates the STING pathway resulting in production of SASP (Glück et al., 2017). Dysregulation and continuous secretion of IL-6 leads to autoimmune disorders and chronic inflammation. Chronic inflammation has been implicated in numerous morbidities with one of the relatively common ones being Arthritis. Links between IL-6 and arthritis are clear and as a consequence Tocilizumab, a humanized anti-IL-6 receptor antibody was FDA approved to treat inflammation-induced arthritis (Grupp et al., 2013).

Since the first studies that reported deleterious effects of senescent cells *in vivo*, efforts have been made to ameliorate senescence-associated pathologies. The first study of this kind used a drug activated FKBP–Caspase-8 fusion protein to induce apoptosis and was driven by a p16 promoter, which is strongly upregulated in senescent cells (Alcorta et al., 1996; Wong and Riabowol, 1996). This removal of senescent cells was shown to increase health span and delay age-related disorders in a progeroid model of accelerated mouse aging (Baker et al., 2011). This led to the later discovery of the first “senolytic” compounds that can selectively target and eliminate senescent cells: Quercetin and Dasatinib (Zhu et al., 2015). Another study found that the antiapoptotic protein BCL2 that is upregulated in senescent cells to block apoptotic cell death can be targeted by inhibitors such as Navitoclax to deprotect and eliminate senescent cells (Zhu et al., 2016). A Dasatinib- Quercetin combination is now in Phase 2 trials after Phase 1 results showed the potential to decrease the senescent cell burden in human patients with diabetic kidney disease (Hickson et al., 2019). At present there are over a dozen candidate senolytics undergoing clinical trials in varying phases, and the development of increasingly sensitive methods to identify senescent cells (González-Gualda et al., 2020) should allow accurate determination of the efficacy of promising senolytics. A, recent 2020 study used constitutively expressed knock-in system that targets p16^High^ cells to show that removal of particular population of senescent cells could be detrimental. The cell population identified in this study was the p16^High^ vascular endothelial cells in liver sinusoids (LSECs). With age, this particular population of cells are increasingly relied on for detoxification and are not replaced upon removal (Grosse et al., 2020). However, the study also showed that while administration of senolytic cocktail Dasatinib + Quercetin removed p16^High^ macrophages, it did not affect the p16^High^ LSECs. Hence going forward, much more work needs to be done regarding understanding the deleterious/beneficial functions of crucial sub-populations of senescent cells, especially with novel senolytics already in clinical trials.

### Senescence in Development: Contributions of p53

p53, popularly known as guardian of the genome (Lane, 1992) is an essential tumor suppressor that responds to a variety of stresses by activating numerous pathways that lead to cell cycle arrest, senescence, and apoptosis. The essential nature of p53 is clear by the fact that it is mutated in more than half of the human cancers. p53 induced cell cycle arrest was initially discovered in rat embryo fibroblasts using a temperature sensitive mutant (Martinez et al., 1991). P53 induced cell cycle arrest was found to be mediated by p21 which inhibits CDK 2/4 and results in a G1 cell cycle arrest state (Harper et al., 1993). This prevents phosphorylation of Rb, which binds to E2F1 transcription factor and promotes silencing of E2F1 targets necessary for cell cycle progression. p53 induced cell cycle arrest is critical for senescence and knocking out p21 has been shown to prevent this senescence induction (Brown et al., 1997).

Senescence is now appreciated to promote organismal aging and cancer development through effects of the p53 tumor suppressor. This idea was solidified by knockout studies of p53 in mice that showed a lack of developmental defects with the main cause of mortality arising from spontaneous tumor formation (Donehower et al., 1992). However, other studies at the time such as knockout of MDM2 that functions to tag p53 for degradation had shown early embryonic lethality due to unchecked p53 activation (Jones et al., 1995). Further studies with mutated forms of p53 in mice which results in CHARGE syndrome, a complex mixture of eye, heart, nasal cavity, growth, genital, and hearing abnormalities, have suggested that precise spatio/temporal regulation of Tp53 is essential for normal development (Nostrand et al., 2014). The nearly normal development of Tp53-null mice could be due to compensatory roles by other p53 family proteins such as p63 and p73. This is consistent with studies in *Xenopus laevis* which lacks the p63 and p73 homologs, where p53 knockout results in severe gastrulation defects and early lethality (Wallingford et al., 1997). While p53 knockout alone does not result in embryonic lethality in mice, p53-deficient female mice embryos show an 8-23% occurrence of exencephaly due to failed neural tube closure (Armstrong et al., 1995; Sah et al., 1995). Developmental defects of the eye, upper incisor tooth formation, and polydactyly of the hind legs were also seen at high occurrence in p53 null mice. Lower fertility is also observed in p53 knockout mice, due to abnormalities in spermatogenesis or embryonic implantation difficulties (Beumer et al., 1998; Hu et al., 2007). p53 is also essential for proper differentiation and development of specific tissues (Molchadsky et al., 2010). p53 fulfills this role though regulating genes required for differentiation programs of neuronal cells, osteoblasts, myocytes, adipocytes and B cells, ensuring proper timing and function of differentiation and development (Molchadsky et al., 2010). p53 clearly plays a functional role in development, however, the described phenotypic abnormalities are less crucial to sustaining life than expected by p53’s essential role as a tumor suppressor (Jain and Barton, 2018).

### Senescence in Development: Contributions of p21

p21 is a cyclin-dependent kinase inhibitor and one of the major effectors of p53. However, p21 can also be activate independently of p53 especially in developmental senescence. p21 binds to PCNA resulting in G1/G2 cell cycle arrest (Luo et al., 1995; Cayrol et al., 1998). While earlier studies suggested p21acts solely as a tumor suppresser, recent studies also suggest that p21 can promote oncogenicity under certain conditions (Roninson, 2002). The CDK inhibitory domain and PCNA binding domains of p21 are responsible for its main growth inhibition characteristics (Chen et al., 1995; Luo et al., 1995). CDK2 inhibition by p21 results in prevention of RB phosphorylation which is necessary for E2F activation and leads to cell cycle arrest (Harper et al., 1993). A variety of structures such as apical ectodermal ridge (AER), neural roof plate, mesonephros, and inner ear endolymphatic sac show certain hallmarks of senescence during the course of normal mammalian embryonic development (Muñoz-Espín et al., 2013; Storer et al., 2013). This developmentally programmed senescence is p21 dependent and unlike other types of senescence such as DNA- damage induced senescence, it is not mediated by p53, but rather regulated by the TGF-β/SMAD and PI3K/FOXO pathways. Developmentally programmed senescence is mainly involved with tissue remodeling, which is executed by macrophages that infiltrate and clear senescent cells. In addition to playing a role in inducing cell cycle arrest and senescence, another major role of p21 in development is the prevention of apoptosis. This is partly due to upregulated anti-apoptotic BCL-2 protein family members in senescent cells (Chang et al., 2016; Zhu et al., 2016). In the mesonephric tubules of p21 null mice embryos, developmentally programmed senescence halts, however, induction of apoptosis takes place the following day which also results in macrophage infiltration followed by clearance of these cells (Muñoz-Espín et al., 2013; Storer et al., 2013). This has led some to suggest that apoptosis and senescence are mutually compensatory mechanisms in mammalian embryonic development, despite being temporally distinct. However, apoptosis does not appear to fully compensate for the absence of developmental senescence. p21 induced senescence plays an important role in the regression of the dorsoventral vaginal septum in female mice. In p21 null female mice, an impaired development of Wolffian duct leads to increased incidence of vaginal septa and decreased fertility (Muñoz-Espín et al., 2013; Storer et al., 2013), suggesting an indirect effect of decreased senescence during development. It will be interesting to see whether future studies confirm the role of p21 induced senescence in other aspects of mammalian development.

### Senescence in Development: Contributions of Rb

Retinoblastoma is a tumor suppressor which plays a which plays a pivotal role in cell cycle regulation and tumor progression. The retinoblastoma gene was the first tumor suppressor to be cloned and was a pivotal moment in the field of cancer genetics (Lee et al., 1987). Rb was identified as the protein mutated in the childhood cancer retinoblastoma, and subsequently found to act in a recessive manner, requiring both copies of the *Rb* gene to be mutated to promote aberrant cell growth (Knudson, 1971). Rb’s role in cell proliferation was first established by its discovery as a primary target of DNA tumor virus oncoproteins (Whyte et al., 1988). Another important discovery was that overexpression of Rb caused a G1 cell cycle arrest (Goodrich et al., 1991). This was later shown to be a result of Rb’s interaction with the E2F family of transcription factors (Dyson, 1998). Rb/E2F pathway functions by recruiting chromatin remodeling factors such as histone deacetylases (HDACs) to repress gene expression (Luo et al., 1998). This was shown to be negatively regulated by CDK mediated phosphorylation of Rb which prevents its binding with E2Fs and HDACs (Harbour and Dean, 2000). Rb tumor suppressor is the primary effector of senescence. Once Rb is fully activated during senescence, it has been speculated that cell cycle arrest becomes permanent and can no longer be reversed by inactivation of Rb or p53. Rb was found to be activated in senescent cells and its enforced expression was found to induce senescence (Lee et al., 2000; Ferbeyre et al., 2002). Moreover, senescence induction by ras oncogene and DNA damaging agents is prevented by adenovirus E1A which targets Rb (Lowe and Ruley, 1993; Serrano et al., 1997). Cells lacking Rb also fail to achieve senescence *in vitro* (Dannenberg et al., 2000; Sage et al., 2000). Knockout or homozygous mutation of the *Rb* gene is embryonically lethal, blocking development between days 13.5 and 15.5 in mice (Jacks et al., 1992). Defects prior to death of the mice include an increase in the number of immature nucleated erythrocytes, inhibition of hepatic erythropoiesis, ectopic proliferation, and apoptosis especially in the hindbrain and death of spinal ganglia (Clarke et al., 1992; Jacks et al., 1992; Lee et al., 1992). The lethal developmental defects of Rb ablation in neurogenesis and hematopoiesis are reversed by insertion of an *Rb* mini transgene into Rb mutant mice. In embryonic development, loss of Rb has also been shown to cause extensive proliferation of trophoblast cells, damage to the normal labyrinth architect of the placenta, and a decline in vascularization and functional placental transport (Wu et al., 2003).

Retinoblastoma not only plays a role in mammalian development but is also crucial in preventing cancer throughout the lifecycle. Inactivation of Rb can occur through phosphorylation, mutation or viral oncoprotein binding leading to malignant phenotypes (Giacinti and Giordano, 2006). Tumors such as osteosarcomas, small cell-lung carcinomas, breast carcinomas, bladder carcinomas, and glioblastomas have been identified to lack functional Rb, indicating homozygous gene mutations or gene absence contributes to the development of these cancers (Yokota et al., 1988; Horowitz et al., 1990; Venter et al., 1991; Borg et al., 1992; Nielsen et al., 1997). The antagonistic pleiotropy theory of aging described initially by Williams (1957) states that early in life, genes are selected for that increase the organism’s fitness, however, they have deleterious effects later in life, contributing to the decline in function described as aging. Apoptosis and senescence have both been thought to display antagonistic pleiotropy through their ability to suppress tumor growth and cancer, but consequently promote an aging phenotype (Campisi, 2003).

## Apoptosis

Although the process of programmed cell death was first described by Carl Vogt in 1842 while studying tadpole development, the term apoptosis was first used by Kerr et al. (1972) to describe controlled, programmed cell death, and with cells exhibiting distinct changes in morphology.

Apoptosis plays a crucial role in clearing redundant, irregular or dangerous cells, while regulating growth, development and the immune response. Cells undergoing apoptosis form apoptotic bodies, through condensation of their cytoplasm and nucleus, DNA fragmentation, loss of cell-cell adhesion, and blebbing of the cell membrane (Kerr, 1971). Apoptotic bodies are quickly phagocytosed by macrophages or neighboring cells, resulting in relatively low levels of inflammation, in contrast to necrosis (Reed, 2000). Once Apoptosis is initiated, it is carried out through the activation of a caspase-cascade, a series of cystine-aspartic proteases (D’Arcy, 2019). The caspases selectively cleave target proteins, either activating or inactivating them to promote cell death (Hengartner, 2000) in response to cell damage and during development.

### Apoptotic Pathways

**FIGURE 2:**
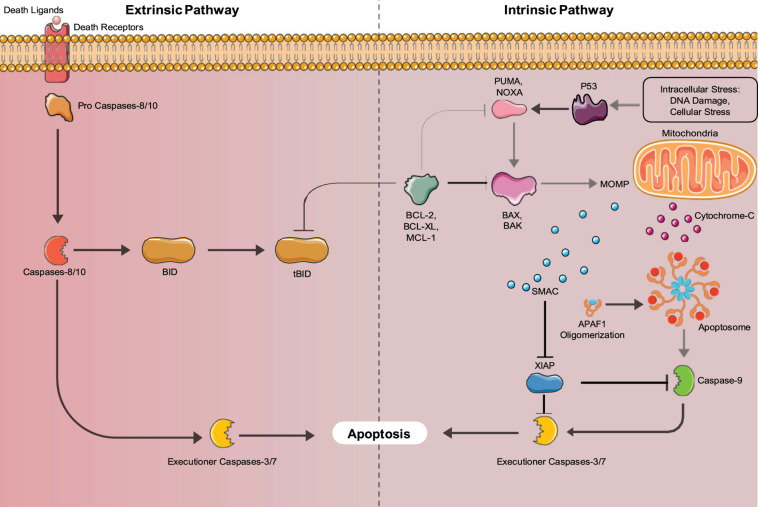
Intrinsic and Extrinsic Apoptosis pathways: Apoptosis can be mediated by either the intrinsic (mitochondria-mediated) pathway or extrinsic (death-receptor mediated) pathways. Both pathways converge upon executioner caspases (Caspase-3 and 7) that induce apoptosis.

There are two major pathways used to activate the caspase cascade, the death receptor-mediated (extrinsic) pathway, and the mitochondria-mediated (intrinsic) pathway shown in Figure 2. In non- apoptotic cells, caspases exist in an inactive zymogen or procaspase form (Budihardjo et al., 1999). The extrinsic pathway activates the caspase cascade, through binding of death ligands to the tumor necrosis factor superfamily (TNF) of cell surface death receptors. Fas, TNF receptor 1 (TNFR1), Death receptor 3 (DR3), DR4/TNF-related apoptosis-inducing ligand receptor 1 (TRAIL-R1), and Dr5/TRAIL-R2 are mammalian death receptors involved in inducing extrinsic apoptosis (Ashkenazi and Dixit, 1998). Each death receptor is able to bind a specific death ligand; Fas binds FasL, TNFR1 binds TNF, DR3 binds Apo3L, and DR4 and DR5 both bind Apol2. The Fas and TNFR1 death receptors both contain a death domain (DD) that they use to recruit adaptor molecules upon ligand binding. Fas-associated protein with death domain (FADD) and TNF- receptor associated death domain (TRADD) bind with their complementary death domain (DD) to Fas and TNFR1, respectively. The cytoplasmic side of the death receptor and it’s corresponding adaptor protein make up the death-inducing signal complex (DISC) (D’Arcy, 2019). The adaptor proteins also contain a death effector domain (DED) that is responsible for recruiting procaspase- 8 and procaspase-10 through their own DED’s to the DISC for activation by proteolytic cleavage. Two separate pathways are regulated by active initiator caspases-8 and -10. The initiator caspases cleave the downstream executioner caspases, caspase-3, caspase-6, and caspase-7, activating and allowing them to transform the cell into an apoptotic body (Singh et al., 2019). Caspase-8 can also activate the intrinsic pathway involving mitochondria, through cleavage of BID, which initiates apoptosome formation, activating caspase-9, thus activating the executioner caspases.

The intrinsic apoptosis pathway is activated by stimuli such as irradiation, chemotherapy, deprivation of cytokines, and growth factors or DNA damage (Igney and Krammer, 2002). These stimuli cause the increased transcription and/or posttranslational modification of BH3-only proteins (BIM, PUMA, BID, BMF, BAD, BIK, NOXA, and HRK) that suppress the activity of cell survival BCL-2 proteins (Aubrey et al., 2017). Inhibition of the pro-survival proteins allows the release of BCL-2 family member proteins BAX and BAK that function to produce mitochondrial outer membrane permeabilization (MOMP), allowing the release of cytochrome C, and SMAC/DIABLO into the cytosol. Cytochrome C binds to apoptotic protease activating factor 1 (APAF1) forming the apoptosome, where caspase-9 is activated (Igney and Krammer, 2002). Active caspase-9 cleaves and activates the executioner caspases (caspases-3,6,7) that induce the hallmark characteristics of apoptotic cells. The SMAC/DIABLO protein functions to inhibit the inhibitors of apoptosis proteins (IAPs), allowing apoptosis to occur through both pathways (Igney and Krammer, 2002). Additionally, unresolved Endoplasmic Reticulum (ER) stress can lead to the induction of apoptosis. ER stress is caused by increased rates of misfolded proteins above the load capable of being handled by chaperones including heat shock proteins (Tabas and Ron, 2011). In mammals, misfolded proteins activate the ER-specific unfolded protein response (UPR), which contains three branches that are transduced by upstream signaling molecules, IRE1, PERK, and AFT6 that sense ER stress (Tabas and Ron, 2011). If the UPR fails to restore proper ER function it will switch to a pro-apoptotic function (Szegezdi et al., 2006). IRE1, PERK, and AFT6 all induce the transcription of CHOP (Szegezdi et al., 2006). IRE1 also recruits apoptosis signal-regulating kinase 1 (ASK1), which signals JNK, a protein kinase (Szegezdi et al., 2006). JNK and CHOP suppress the anti-apoptotic properties of BCL-2 proteins (Szegezdi et al., 2006). Bax and Bak are then expressed and subsequently activate the caspases that directly initiate apoptosis (Szegezdi et al., 2006). ER stress also induces senescence through the AFT6 branch of the UPR (Druelle et al., 2016). ATF6 was shown by Druelle et al. (2016) in normal human dermal fibroblasts to be important for controlling the increase in SA-β-Gal activity, the cell morphological changes associated with senescence and the ER expansion associated with the senescent phenotype (Druelle et al., 2016). AFT6 upregulates the COX2/PGE_2_ intracrine pathway, which contributes to the induction and maintenance of the senescent phenotype (Cormenier et al., 2018).

Another form of cell self-destruction is autophagy. In mammals, autophagy is the mechanism where cytosolic components of a cell are delivered to the lysosome for degradation and the products can be recycled and used as building blocks for biosynthetic reactions or for energy production (Mizushima and Komatsu, 2011). Interestingly, autophagy not only plays a role in cell destruction, but also functions in maintaining stemness. García-Prat et al. (2016) demonstrated that as mice age their muscle stem cells lose their autophagy ability, causing the accumulation of damaged cellular components, with cells senescing and losing their properties of stemness (García-Prat et al., 2016; Tabas and Ron, 2011; Szegezdi et al., 2006; Druelle et al., 2016; Cormenier et al., 2018; Mizushima and Komatsu, 2011).

### Apoptosis in Development

**FIGURE 3:**
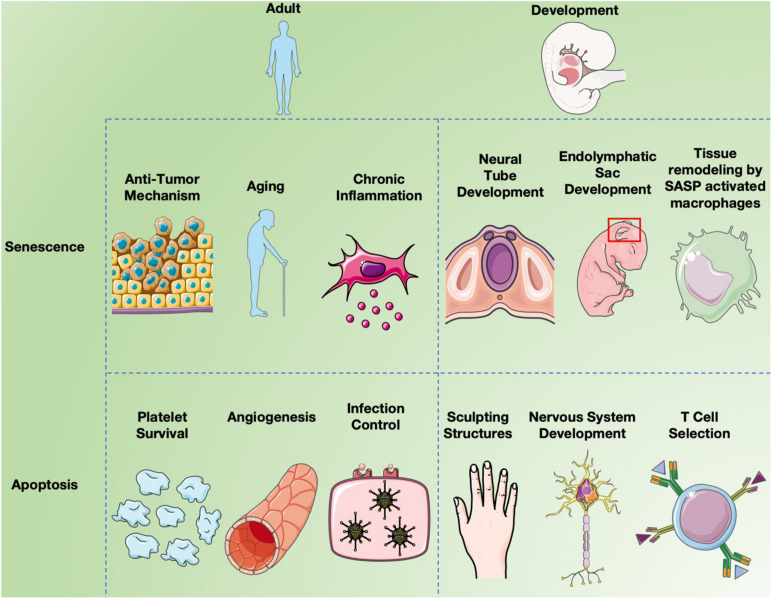
Senescence and apoptosis in development and aging. Cell senescence is clearly a major factor in inducing chronic inflammation by fragmented DNA activating the innate immune response via cGAS- STING, resulting in senescent cells secreting a combination of factors collectively called the SASP (Hopfner and Hornung, 2020). Inflammation induces many aspects of aging, but cell senescence is also believed to serve as a “first line of defense” against cancer since it limits cell replication (Campisi, 2003). While apoptosis long been known to affect different aspects of both development and aging, recent studies have implicated programmed cell senescence in tissue remodeling of different types (Muñoz-Espín et al., 2013; Storer et al., 2013; Zhao et al., 2018).

In mammals and other vertebrates, apoptosis is crucial for the formation and maintenance of tissue patterning (Meier et al., 2000; Zakeri et al., 2015) and is important for the development of the central nervous system (Naruse and Keino, 1995; Washausen et al., 2018) in a segmental manner (Lumsden et al., 1991; Knabe et al., 2004) including the optic system (Knabe and Kuhn, 1998; Knabe et al., 2000) as noted in Figure 3. The formation of the neural tube from the neural plate is thought to be the first critical developmental stage in the CNS aided by apoptosis. Caspase 3 and 9 knockout mice display defective brain development due to excess neuroepithelial cells, while a knockout of caspase 8 affects cardiac muscle development and erythropoiesis (Kuida et al., 1996, 1998; Hakem et al., 1998; Varfolomeev et al., 1998). Caspase 3 mutant mice are born at a lower fequencey than expected by mendelian genetics and most die shortly after birth, due to caspase 3’s required role in brain development (Woo et al., 1998). Similarly, caspase 9 deficient mice die prenatally due to their inability to activate the caspase 3 executioner caspase, resulting in perturbations of brain morphology. Caspase 8 knockout mice are also embryonic lethal as caspase 8 absence obstructs apoptosis induction by TNF death receptors resulting in abnormal phenotypes. Additionally Caspase-8C362S/C362S mice are also embryonic lethal (Fritsch et al., 2019). Mice lacking Apaf-1, a key component of the apoptosome, showed reduced apoptosis in the developing brain, causing hyperproliferation of neurons and craniofacial irregularities (Yoshida et al., 1998). Apaf-1 deficient mice also had persistence of interdigital webs, abnormalities of the lens and retina, and an inability to activate caspase 3, leading to death at embryonic day 16.5 (Cecconi et al., 1998). Less than 10% of mice deficient in Bak and Bax survived and had birth defects such as imperforate vaginal canals, interdigital webbing and excess brain cells (Lindsten et al., 2000). As well, only 2% of Bok, Bax and Bak knockout mice survived to weaning with non-lethal abnormalities (Ke et al., 2018). These observations confirm the importance of apoptosis for normal development.

Apoptosis plays a role in sculpting structures in vertebrates, through the deletion of cells between developing digits as well as deletion of cells to form hollow structures (Jacobson et al., 1997). Structures are formed during development that later do not serve a purpose and are thus removed by apoptosis such as removal of the rudimentary lateral line system of aquatic vertebrates, from mice (Washausen and Knabe, 2018). These structures also include ones needed by only one sex, vestigial structures, and structures only needed for certain developmental stages. Apoptosis eliminates pronephric tubules in mammals, but not in amphibians or fish where they form functional kidneys. The Wolffian and Mullerian ducts are only required in male and female reproductive systems, respectively, and are eliminated by apoptosis in the opposite sex (Meier et al., 2000). Cells may be over produced during embryonic development, and apoptosis helps to control this. During mammalian development, apoptosis also aids in quality control, through removal of irregular, non-functional or dangerous cells, and preventing birth defects and cancer (Jacobson et al., 1997).

Different apoptosis regulatory proteins are expressed variably throughout mammalian development. In early embryonic development of mice, BCL-2 is extensively expressed in all germ layers, but its expression declines during maturation. Norvak and Korsmeyer found that in endodermal epithelial cells of the lung bud, Bcl-2 is expressed in a proximal to distal gradient at E12.5 and increases by day E18.5. In mesodermal tissues, the uterine bud and metanephric cap of the kidney, Bcl-2 is expressed at E 12.5 (Novack and Korsmeyer, 1994). Retinal neuroepithelial cells derived from the ectoderm show uniform expression of Bcl-2 up until differentiation, then a topographic distribution is established that persists throughout maturation. As predicted, Bcl-2 is only present in regions of cell survival such as the digital zones of the developing limb, and its expression is absent in the interdigital zones where cells die before the end of fetal development. Bcl-2 expression decreases in the central nervous system following neural tube formation but remains highly expressed in the peripheral nervous system (Merry et al., 1994). Caspase 3 has been shown to be highly expressed in the brain during embryonic and early post-natal development, but this declines at 4 weeks postnatal and its absence remains throughout aging (Shimohama et al., 1999). These changes in expression correlate well with the removal and maintenance of various structures during development. Table 1 details a summary of developmental phenotypes of transgenic mice associated with apoptosis/senescence related genes.

**TABLE 1:** Summary of transgenic mice developmental phenotypes with knockout or knock-in of key genes involved in apoptosis and senescence.

KO mice type	Pathway effected	Developmental phenotype	References
p21^–/–^	Senescence	Impaired development of Wolffian duct, apical ectodermal ridge, and decreased fertility in female mice	Muñoz-Espín et al., 2013; Storer et al., 2013
p53^–/–^	Senescence	Tumour formation 8–23% of female mice develop exencephaly. Defects in eye, upper incisor tooth, and polydactyl of hind legs. Low fertility	Donehower et al., 1992; Armstrong et al., 1995; Sah et al., 1995; Beumer et al., 1998; Hu et al., 2007
Rb^–/–^	Senescence	Embryonic lethal	Jacks et al., 1992
Ing1^–/–^	Apoptosis	Reduced size, increased Bax expression, and DNA damage induced apoptosis	Kichina et al., 2006; Coles et al., 2007
Bax^–/–^ and Bak^–/–^	Apoptosis	Less than 10% survive to weaning, and have birth defects including imperforate vaginal canal, interdigital webbing, and excess brain cells	Lindsten et al., 2000
Bok^–/–^, Bak^–/–^, and Bax^–/–^	Apoptosis	2% survived to weaning with non-lethal abnormalities	Ke et al., 2018
Caspase-3^–/–^	Apoptosis	Excess neuroepithelial cells, death shortly after birth	Kuida et al., 1996; Hakem et al., 1998; Woo et al., 1998
Caspase 9^–/–^	Apoptosis	Defective brain development, death shortly after birth	Kuida et al., 1998
Caspase-8^–/–^	Apoptosis	Embryonic lethal	Varfolomeev et al., 1998
Caspase-8^*C3626/C3626*^	Apoptosis	Embryonic lethal	Fritsch et al., 2019
Apaf-1^–/–^	Apoptosis	Embryonic lethal	Cecconi et al., 1998; Yoshida et al., 1998

*Apoptosis and senescence are clearly crucial processes involved in development, as these knockouts show birth defects or premature death.*

## Dual Functions of p53, Rb, and ING1 in Senescence and Apoptosis

The p53 protein can induce senescence followed by apoptosis in cells with unrepairable damage (Amundson et al., 1998). p53’s ability to activate apoptosis was clear when thymocytes in p53 knockout mice were shown to be resistant to apoptosis induced by radiation or other stimuli that cause DNA damage (Clarke et al., 1993; Lowe et al., 1993). Cells undergoing p53-dependent apoptosis normally proceed through the intrinsic pathway. The BH3-only proteins PUMA and NOXA are direct targets of p53 for upregulated transcription and expression leading to the induction of apoptosis (Oda et al., 2000; Nakano and Vousden, 2001). BAX and BID are also direct targets of p53, and their expression is upregulated during p53-dependent apoptosis (Toshiyuki and Reed, 1995; Sax et al., 2002). In addition to its function as a transcription factor, p53 acts also acts in the cytoplasm, binding to anti-apoptotic, pro-cell survival BCL-2 proteins (BCL-2 and BCL-x), which releases the pro-apoptotic proteins, BAX and BAK to carry out MOMP, and subsequently the rest of apoptosis (Schuler and Green, 2005). APAF-1, the protein required for apoptosome formation is also a transcriptional target of p53 during apoptosis (Moroni et al., 2001). In cells undergoing apoptosis induced by hypoxia, p53 has been found to target and upregulate the death receptor Fas (Liu et al., 2006). The p53 apoptosis pathway is also activated by the loss of Rb function, to remove cells lacking this protective anti-tumor mechanism (Morgenbesser et al., 1994). p53 mediated apoptosis might also act to prevents birth defects (Norimura et al., 1996). When pregnant mice with p53^–/–^ embryos were exposed to ionizing radiation, there was a high rate of birth defects (70%) and low rate of embryonic lethality (7%) compared to wildtype p53^+/+^ embryos that had 60% embryonic death, and only 20% of offspring with birth defects. p53 is thought to prevent birth defects through eliminating abnormal cells, however, if too many cells are removed through apoptosis the embryo dies.

Depending upon the stage of the cell cycle, Rb can have an anti-apoptotic or pro-apoptotic function (Ianari et al., 2009). Rb plays an anti-apoptotic role in cells present in G0/G1 (non-proliferating), by suppressing EF2 transcription factors, which typically contribute to apoptosis progression by transcriptionally activating p73 and caspase-7 (Iaquinta and Lees, 2007). In contrast, in proliferating cells, Rb acts as a pro-apoptotic regulator in the presence of stressors that cause DNA- damage. Under these conditions Rb is phosphorylated resulting in the formation of the pRB-E2F1- P/CAF complex which is active at pro-apoptotic gene promoters (Ianari et al., 2009). Figure 3 shows an overview of both post and pre developmental roles senescence and apoptosis plays in mammalian systems.

The ING (“INhibitor of Growth”) tumor suppressors are also capable of inducing both senescence and apoptosis, largely by their effects on the p53 and Rb pathways. The first report of ING1 affecting apoptosis came from study of P19 teratocarcinoma cells where serum starvation caused increased expression of ING1 and consequent induction of apoptosis that appeared to occur in collaboration with c-Myc (Helbing et al., 1997). The ability of ING1 to induce apoptosis in response to UV-induced DNA damage was dependent upon the PCNA-interacting Protein (PIP) motif of ING1 (Scott et al., 2001) while the ability of both ING1and ING2 to efficiently induce apoptosis via p53 depends largely on their polybasic regions that bind the bioactive stress-induced phospholipid phosphatidyl inositol 5′-monophosphate (Gozani et al., 2003). Both ING1 and ING2 are stoichiometric members of the Sin3a histone deacetylase complex (Doyon et al., 2006) and directly affect p53 acetylation at lysine residues 373 and/or 382 (Kataoka et al., 2003), activating it as a growth inhibitor and inducer of apoptosis. ING1 was also shown to be required for the full ability of p53 to block cell growth (Garkavtsev et al., 1998) and this was later determined to be a consequence of ING1 stabilizing p53 by multi/mono-ubiquitination, preventing the polyubiquitination and subsequent proteosome- dependent degradation of p53 (Thalappilly et al., 2011). ING1 also promoted p53 accumulation in response to oncogenic stress (Abad et al., 2007). Like p53, ING1 can promote apoptosis through effects at the mitochondria of fibroblasts by interacting with Bax (Bose et al., 2013) and in melanoma cells in response to UV induced DNA damage, ING1 promoted p53-induced Bax expression to alter mitochondrial membrane potential and induce apoptosis (Cheung and Li, 2002). Links between ING proteins and cell senescence were also apparent since knockdown of either ING1 (Garkavtsev and Riabowol, 1997) or ING2 (Pedeux et al., 2005) that are both components of the Sin3a HDAC complex extends cell replicative lifespan, delaying senescence. In contrast, the ING1a splicing variant of ING1 that increases ∼10-fold during cell senescence (Soliman et al., 2008) induces senescence much more rapidly than other stresses (Rajarajacholan and Riabowol, 2015) via disrupting endocytosis that activates the Rb/p16/p57 pathway. ING1a expression imposes most of the hallmarks of cell senescence including growth arrest, senescence-associated b-Galactosidase expression, increased p16 and Rb, senescence-associated heterochromatic foci and phenotypic changes resembling replicative senescence (Rajarajacholan et al., 2013). Knockout of Ing1 results in mice with reduced overall size and body weight, increased turnover of thymocytes, behavioral abnormalities and increased sensitivity to ionizing radiation, and consequently a shortened lifespan. While there were no other obvious developmental abnormalities, an earlier and higher incidence of lymphomas was also reported (Kichina et al., 2006). While Ing1 deficient mouse embryonic fibroblasts (MEF) have an increased growth rate, this was also sustained in p53 deficient MEFs suggesting a p53 independent role in cell proliferation. Ing1 knockout also induces Bax expression and promotes DNA damage-induced apoptosis independent of p53 (Coles et al., 2007).

## Summary

The p53, Rb, and ING tumor suppressors all affect cell growth, apoptosis and senescence, and in doing so at different stages of cell lifespan, exhibit a degree of antagonistic pleiotropy. ING1 potentiates the activity of both p53 and Rb as inhibitors of growth and the p53 and Rb pathways show crosstalk through the CDK inhibitors p16 and p21. While p53, Rb, and ING1 play central roles in maintaining normal cell cycle progression and guard against unregulated cell growth to inhibit oncogenesis, these proteins later take on a significantly stronger role in growth inhibition, ultimately blocking cell growth fully in the state of replicative senescence.

## Author Contributions

EW and HT wrote the first draft. HT produced the figures. KR edited the manuscript and obtained funding. All authors contributed to the article and approved the submitted version.

## Conflict of Interest

The authors declare that the research was conducted in the absence of any commercial or financial relationships that could be construed as a potential conflict of interest.
